# Supplementary material for: Integrating Basic and Clinical Sciences Using Point-of-Care Renal Ultrasound for Preclerkship Education
Source: MedEdPORTAL. 2020 Dec 9;16:11037. doi: 10.15766/mep_2374-8265.11037 (PMC7732135; doi:10.15766/mep_2374-8265.11037)
Supplement: Supplementary file 1 — Hands-on Session Setup Instructions.docxPractical Session Room Setup.docxHands-on Session Instructor Guidelines.docxOSCE Checklist Renal.docxNote for Ultrasound Models.docxMS1 Renal Lecture With Presenter Notes.pptxPremodule Survey.docxPostmodule Survey.docx [file mep_2374-8265.11037-s001.zip › A. Hands-on Session Setup Instructions.docx]

**Hands-on Session Set-Up Instructions**

This document will review the resources (space, station components, and faculty) required for the second component of the module—the point-of-care ultrasound (POCUS) hands-on session.

1. Space:

The space needed for the session depends on the size of the class and resources. We used one large classroom to accommodate four individual teaching stations. We had 31 participants total over 2 sessions with 15-16 students in each session. Thus, we divided the students in each session into 4 groups, resulting in 3-4 students with 1 instructor and 1 standardized patient at each teaching station.

1. Station Components:

Each station consists of a stretcher and a portable handheld ultrasound connected to an iPad (Appendix B). The compact, portable handheld unit should have capability of scanning with either low-frequency curvilinear or phased-array transducer settings. These are ideal for imaging abdominal structures. The handheld ultrasound must also have Doppler capabilities.

Each station should have a patient gown for the standardized patient and an adequate amount of ultrasound gel and paper towels to remove the gel after each scan. The workshop objectives and OSCE checklist should be written on a dry-erase board or large pad adjacent to each station, to be reviewed at the beginning and end of each session.

1. Faculty Required:

The goal of the hands-on session is to have a reasonably low student-to-instructor ratio (maximum of 4- or 5-students to one instructor), which allows opportunity for hands-on practice by the students and personalized direct instruction. The students at each station should rotate clock-wise every 20 minutes, so that by the end of the session, they have scanned three separate standardized patients of various body types under the instruction of three separate instructors with varying teaching styles.

1. Standardized Patients (SPs):

The SPs can either be male or female, but typically are of thin-to-average size frame with a body mass index < 30, since body habitus affects the depth at which ultrasound visualization of organs can be acquired. Since this session is usually the students’ first time using bedside ultrasound, high-resolution images are desired. In addition to treating the SPs professionally, they should be provided with instructions and expectations in advance of the session (Appendix E).
