# Supplementary material for: Integrating Basic and Clinical Sciences Using Point-of-Care Renal Ultrasound for Preclerkship Education
Source: MedEdPORTAL. 2020 Dec 9;16:11037. doi: 10.15766/mep_2374-8265.11037 (PMC7732135; doi:10.15766/mep_2374-8265.11037)
Supplement: Supplementary file 1 — Hands-on Session Setup Instructions.docxPractical Session Room Setup.docxHands-on Session Instructor Guidelines.docxOSCE Checklist Renal.docxNote for Ultrasound Models.docxMS1 Renal Lecture With Presenter Notes.pptxPremodule Survey.docxPostmodule Survey.docx [file mep_2374-8265.11037-s001.zip › B. Practical Session Room Setup.docx]

**Hands-on Module Room Set-up**

Standard examination table with standardized patient

Student 1

Student 2

Student 3

Student 4

Instructor with iPad

iPad

Handheld transducer

Table with checklist, ultrasound gel, and napkins
