# Supplementary material for: Integrating Basic and Clinical Sciences Using Point-of-Care Renal Ultrasound for Preclerkship Education
Source: MedEdPORTAL. 2020 Dec 9;16:11037. doi: 10.15766/mep_2374-8265.11037 (PMC7732135; doi:10.15766/mep_2374-8265.11037)
Supplement: Supplementary file 1 — Hands-on Session Setup Instructions.docxPractical Session Room Setup.docxHands-on Session Instructor Guidelines.docxOSCE Checklist Renal.docxNote for Ultrasound Models.docxMS1 Renal Lecture With Presenter Notes.pptxPremodule Survey.docxPostmodule Survey.docx [file mep_2374-8265.11037-s001.zip › C. Hands-on Session Instructor Guidelines.docx]

**Renal Point-of-Care Ultrasound Small Group Facilitator’s Guide**

Flow and timeline of activities

*Pre-Session (15-20 minutes prior to the session)*

1. Prepare and ensure room set-up including point-of-care ultrasound (POCUS) machines, standardized patient, and audiovisual equipment (iPad, ultrasound transducer with iPad application) (Appendix B)

*Session (120 minutes)*

1. Didactic session with PowerPoint presentation (Appendix F) *(30 minutes)*
2. Small group hands-on scanning session *(90 minutes)*

a. One facilitator per every 4 students *(20 minutes per group)*

Expectations for small group facilitator

*Pre-Session*

1. Provide orientation and expectations for the standardized patient (Appendix E)
2. Pre-perform the POCUS checklist on the standardized patient so you are familiar with the anatomy on the standardized patient before students arrive

*Session*

1. Ensure every student is able to demonstrate acquisition of each image listed on checklist
2. Guide students to find and optimize POCUS images by having them place the ultrasound transducer in anatomically correct location, adjust ultrasound settings as needed, and maneuver the position of both ultrasound transducer and position of patient to optimize image quality
3. Ask other students to discuss pathophysiology of specific renal diseases, indications for renal POCUS, and related POCUS findings while they await their turn to perform. Discussion should include:
   1. Renal or ureterovesicular junction stone in a patient presenting with flank pain, lower abdominal/groin pain, and/or hematuria
   2. Hydronephrosis in a patient with signs/symptoms of renal stone or urinary retention
   3. Renal mass or cyst in patient with flank pain, hematuria, and/or new renal dysfunction
   4. Bladder volume in a patient with urinary retention and/or acute suprapubic pain
   5. Bladder mass in a patient with hematuria and/or urinary retention

Tips for helping students

1. Allow students time to acquire images and troubleshoot problems on their own before providing more assistance
2. Allow students to hold and manipulate the transducer independently
3. To troubleshoot poor image quality:
   1. Apply more ultrasound gel
   2. Apply more pressure with ultrasound transducer
   3. Move ultrasound transducer to find optimal anatomic location (may be slightly different for each patient)
   4. Adjust the depth and gain settings on the machine
   5. Adjust patient position by having the patient raise arms or take a deep breath

Suggested FOAMed (Free Open Access Medical Education) resources for renal ultrasound:

1. Society for Academic Emergency Medicine Academy of Emergency Ultrasound (SAEM AEUS): <https://www.saem.org/aeus/education/online-education/aeus-narrated-lecture-series>
2. POCUS Atlas <http://www.thepocusatlas.com>
3. Sonosite: <https://www.youtube.com/playlist?list=PLC94DAEF85E728A0E>
