# Supplementary material for: Integrating Basic and Clinical Sciences Using Point-of-Care Renal Ultrasound for Preclerkship Education
Source: MedEdPORTAL. 2020 Dec 9;16:11037. doi: 10.15766/mep_2374-8265.11037 (PMC7732135; doi:10.15766/mep_2374-8265.11037)
Supplement: Supplementary file 1 — Hands-on Session Setup Instructions.docxPractical Session Room Setup.docxHands-on Session Instructor Guidelines.docxOSCE Checklist Renal.docxNote for Ultrasound Models.docxMS1 Renal Lecture With Presenter Notes.pptxPremodule Survey.docxPostmodule Survey.docx [file mep_2374-8265.11037-s001.zip › D. OSCE Checklist Renal.docx]

**The Visualization of Live Anatomy and Positive Physical Exam Findings**

**Using Ultrasonography**

A Pre-Clinical Undergraduate Medical Education Module for the Renal System

OBJECTIVE STRUCTURED CLINICAL EXAMINATION (OSCE) CHECKLIST

**Views to Acquire Structures Visualized? (Y/N)**

| **Kidney in Sagittal Plane** | Cortex  Medulla  Renal Pyramids  Pelvis  Hepato-renal Space  Spleno-renal Space | \|  \| \| --- \| \|  \| \|  \| \|  \| \|  \| \|  \| |
| --- | --- | --- | --- | --- | --- | --- | --- | --- |
| **Kidney in Transverse Plane** | Cortex  Medulla  Renal Pyramids  Pelvis | \|  \| \| --- \| \|  \| \|  \| \|  \| |
| **Bladder** | Sagittal View  Transverse View  Ureterovesicular Junction  Ureteral Jets  Prostate (in males)  Uterus (in females) | \|  \| \| --- \| \|  \| \|  \| \|  \| \|  \| \|  \| |
