# Supplementary material for: Integrating Basic and Clinical Sciences Using Point-of-Care Renal Ultrasound for Preclerkship Education
Source: MedEdPORTAL. 2020 Dec 9;16:11037. doi: 10.15766/mep_2374-8265.11037 (PMC7732135; doi:10.15766/mep_2374-8265.11037)
Supplement: Supplementary file 1 — Hands-on Session Setup Instructions.docxPractical Session Room Setup.docxHands-on Session Instructor Guidelines.docxOSCE Checklist Renal.docxNote for Ultrasound Models.docxMS1 Renal Lecture With Presenter Notes.pptxPremodule Survey.docxPostmodule Survey.docx [file mep_2374-8265.11037-s001.zip › E. Note for Ultrasound Models.docx]

**Letter to Ultrasound Models**

Greetings!

Thank you very much for agreeing to serve as an ultrasound model for this medical student educational session. Your assistance is very much appreciated. There have been many logistics involved in preparation for this session, so in order for it to progress smoothly, please read the following instructions and pointers beforehand:

1. The module has been carefully designed and scripted within the designated time frame. Once the didactic lectures are completed, the students will make their way to the assigned ultrasound rooms/stations. At this time, please be ready on your respective stretcher in your assigned room.
2. The students know to introduce themselves to you and to show proper professionalism. Please let us know if you encounter any concerns.
3. Ultrasonography requires the use of ultrasound gel placed on the areas of the body to be evaluated. We will have paper towels available for cleaning.
4. The ultrasound scans being performed are not comprehensive, nor can they substitute for a definitive ultrasound ordered by your physician. However, in the rare case that some pathology is uncovered, we will make sure to discuss this with you in private so that you may follow up with your primary physician.

These modules would not be possible without your assistance. Please let us know if you have any questions or concerns. Thank you once again!

Sincerely,
