# Supplementary material for: Integrating Basic and Clinical Sciences Using Point-of-Care Renal Ultrasound for Preclerkship Education
Source: MedEdPORTAL. 2020 Dec 9;16:11037. doi: 10.15766/mep_2374-8265.11037 (PMC7732135; doi:10.15766/mep_2374-8265.11037)
Supplement: Supplementary file 1 — Hands-on Session Setup Instructions.docxPractical Session Room Setup.docxHands-on Session Instructor Guidelines.docxOSCE Checklist Renal.docxNote for Ultrasound Models.docxMS1 Renal Lecture With Presenter Notes.pptxPremodule Survey.docxPostmodule Survey.docx [file mep_2374-8265.11037-s001.zip › H. Postmodule Survey.docx]

**The Visualization of Live Anatomy and Positive Physical Exam Findings Using Ultrasonography**

A Pre-Clinical Undergraduate Medical Education Module for the Renal System

POST-MODULE SURVEY

| **Please indicate your level of agreement with the following statements**: | **Strongly Disagree**  **1** | | **Disagree**  **2** | **Neutral**  **3** | **Agree**  **4** | **Strongly Agree**  **5** |
| --- | --- | --- | --- | --- | --- | --- |
| 1. My curriculum prepares me well for Step 1. |  | |  |  |  |  |
| 2. I would like ultrasound to be integrated into the medical curriculum. |  | |  |  |  |  |
| 3. An understanding of ultrasound will help me take better care of my patients. |  | |  |  |  |  |
| 4. An understanding of ultrasound will help me in my future career (and specialty). |  | |  |  |  |  |
|  | | | | | | |
| *The session content…* |  | |  |  |  |  |
| 5. Allowed for integration of basic science knowledge into a clinical context. |  | |  |  |  |  |
| 6. Was relevant to my learning. |  | |  |  |  |  |
| 6a. Met the learning objectives. |  | |  |  |  |  |
| 7. Was relevant to my preparation for Step 1. |  | |  |  |  |  |
|  | | | | | | |
| *The session helped me…* | | | | | | |
| 8. Better integrate anatomy learning. |  | |  |  |  |  |
| 9. Better integrate physical exam learning. |  | |  |  |  |  |
| 10. Better understand the use of medical imaging in clinical medicine. |  | |  |  |  |  |
| 11. Better understand pathophysiology of disease. |  | |  |  |  |  |
| 12. Learn to use ultrasound for clinical decision-making. |  | |  |  |  |  |
|  | | | | | | |
| *I feel comfortable using ultrasound for…* | | | | | | |
| 13. Delineating anatomy of the renal system. |  | |  |  |  |  |
| 14. Supplementing and confirming positive physical exam findings. |  | |  |  |  |  |
| 15. Supplementing and confirming other medical imaging tests. |  | |  |  |  |  |
| 16. Visualizing the pathophysiology of disease. |  | |  |  |  |  |
| 17. Making clinical decisions for my patients. |  | |  |  |  |  |
|  | | | | | | |
| List 3 things you have gained from this session.  1.  2.  3. | | Comments and Feedback | | | | |
