# Supplementary material for: Integrating Basic and Clinical Sciences Using Point-of-Care Renal Ultrasound for Preclerkship Education
Source: MedEdPORTAL. 2020 Dec 9;16:11037. doi: 10.15766/mep_2374-8265.11037 (PMC7732135; doi:10.15766/mep_2374-8265.11037)
Supplement: Supplementary file 1 — Hands-on Session Setup Instructions.docxPractical Session Room Setup.docxHands-on Session Instructor Guidelines.docxOSCE Checklist Renal.docxNote for Ultrasound Models.docxMS1 Renal Lecture With Presenter Notes.pptxPremodule Survey.docxPostmodule Survey.docx [file mep_2374-8265.11037-s001.zip › _Educational_Summary_Report_11037.pdf]

MedEdPORTAL® | The AAMC Journal of Teaching and Learning Resources

Original Publication

Open Access

Integrating Basic and Clinical Sciences Using Point-of-Care Renal Ultrasound for Preclerkship Education

Stephen Alerhand, MD*, April Choi, MD, Ilya Ostrovsky, MD, Sophia Chen, DO, MPH, Christine Ramdin, PhD, Maria Laboy, MD, Sangeeta Lamba, MD, MS-HPEd

*Corresponding Author: stephen.alerhand@gmail.com

Abstract

Introduction: Point-of-care ultrasound (POCUS) is a valuable asset in bedside clinical care. Undergraduate medical education is increasingly using POCUS as an adjunct tool for teaching anatomy, pathophysiology, and physical exam in an integrated manner. Many medical schools teach content in an organ systems–based format in the preclerkship years. POCUS teaching can be very effectively tailored to specific organ systems. Though pilot curricula for generalized ultrasound education exist, few teach organ systems–based content using POCUS. To address this gap, we designed and implemented an integrated POCUS module to supplement anatomy, pathophysiology, and physical exam teaching in the renal course. **Methods:** The module consisted of (1) a 30-minute didactic lecture introducing students to renal ultrasound technique and image interpretation and (2) a practical hands-on skills session. Pre- and postmodule surveys assessed the efficacy and impact of the curriculum. **Results:** A total of 31 first-year medical students completed the POCUS renal curriculum. A majority reported that the module positively affected their understanding of renal pathophysiology and the physical exam. They also reported increased confidence in using POCUS to detect renal pathology and make clinical decisions. **Discussion:** It was feasible to implement a POCUS curriculum to supplement integrated teaching of renal system concepts in the first year of medical school, and students found POCUS teaching valuable. POCUS provides educators with another tool to integrate basic and clinical sciences with hands-on relevant clinical skills practice in early medical school years.

Keywords
Point-of-Care Ultrasound, POCUS, Ultrasound, Renal, Kidney, Bladder, Anatomy, Physical Exam, Pathophysiology, Clinical Teaching/Bedside Teaching

Educational Objectives

By the end of this module, learners will be able to:

1. Describe the principles of ultrasonography that allow for generation of images on the ultrasound machine's screen.
2. List indications for point-of-care ultrasound (POCUS) in patients presenting with renal and genitourinary complaints.
3. Demonstrate acquisition of POCUS images of the left and right kidneys, bladder, hepatorenal space, and splenorenal space on standardized patients using a handheld ultrasound transducer.
4. Compare POCUS findings with those from other advanced imaging modalities such as computed tomography.
5. Correlate abnormal physical exam findings with associated pathology seen on ultrasonography.
6. Recognize abnormal POCUS findings and describe their associated pathophysiology.

Citation:
Alerhand S, Choi A, Ostrovsky I, et al. Integrating basic and clinical sciences using point-of-care renal ultrasound for preclerkship education. *MedEdPORTAL*. 2020;16:11037.
https://doi.org/10.15766/mep_2374-8265.11037

Introduction

Similar to other advanced imaging modalities, ultrasonography was once performed and interpreted only by trained personnel in the radiology department. The evolution of point-of-care ultrasound (POCUS) has made portable bedside ultrasonography accessible to any medical provider in various settings, and it is a standard of care for many procedures and clinical scenarios. In contrast to the radiology ultrasound, POCUS is a tool for the patient's bedside, to answer a specific question and enable rapid clinical decision-making.

Copyright © 2020 Alerhand et al. This is an open-access publication distributed under the terms of the Creative Commons Attribution license.

Additionally, POCUS is rapidly gaining recognition as an invaluable tool in undergraduate medical education by both educators and medical students.[1] Offering a dynamic view of anatomy as opposed to the static view afforded by traditional imaging, integration of POCUS into preclerkship medical education has been shown to improve students' understanding of anatomy, pathophysiology, and physical exam skills.[2-6] Integration of POCUS has been described in a variety of curriculum designs, ranging from targeted ultrasound sessions incorporated into a specific course to longitudinal training spanning the entirety of medical school.[2,4,7]

Most medical schools now implement organ systems–based curricula during the preclerkship years.[8] POCUS can be easily tailored for integration into the teaching of anatomy, pathophysiology, and physical exam of specific organ systems.[2,6,9,10] We have found ultrasound curricula for teaching abdominal anatomy to preclerkship medical students and scrotal pathology to fourth-year medical students.[11,12] There are also several ultrasound curricula for teaching bedside POCUS for pathological findings and procedures to advanced trainees such as residents.[13-17] However, there is little published literature on teaching POCUS to preclerkship medical students based on an organ system that integrates teaching of anatomy, pathophysiology, and the clinical exam. We address this gap for the renal system in this publication.

In addition to supplementing preclerkship education on the renal system, renal POCUS can also offer students advantages in clinical practice skills as it is frequently used to evaluate for renal pathology such as hydronephrosis and renal masses by various specialties.[18-24] One medical school in Canada found that integration of ultrasound into their renal basic science course helped students achieve course objectives, but their curriculum has not been published.[25] Thus, we share our pilot renal POCUS curriculum for preclerkship medical students that educators can adapt and apply at their own institutions.

Methods

Institutional Review Board Approval

This study was approved by the Institutional Review Board of the Rutgers New Jersey Medical School (Pro2019001951).

Learners

The target learners for this module were preclerkship medical students in the renal organ systems–based course, the last course of the first year of medical school, held from May to June. Prior to this module, students learned about the basic foundations of biochemistry, genetics, microbiology, and pharmacology as well as clinical interviewing and physical exam skills. This was followed by the musculoskeletal, cardiac, and pulmonary organ systems–based courses. The students also received didactics on anatomy, pathophysiology, and clinical management of renal diseases through lectures and team-based learning activities. The students did not have prior exposure to POCUS.

Recruitment of Learners

Participation in this module was voluntary without any prerequisites. Three weeks prior to the module, an informative email was sent to all first-year medical students. The email included a link to an online sign-up sheet where students could register for the module. One week prior to the module, the registrations were binding such that students would have to find a replacement if they could no longer attend the module. The module was offered twice with a cap of 22 students per session. Spots were filled on a first-come, first-served basis.

Setting

The module took place at a clinical skills simulation center that had a conference room and several smaller rooms simulating clinic offices. The conference room had audio/video capabilities and included a projection screen. Each small room included an examination table (Appendix A).

Equipment

The required equipment included ultrasound gel, napkins, a handheld ultrasound transducer, and iPads loaded with the associated ultrasound program that displayed transmitted images from the probe (Appendix B).

Personnel

The module was led by one emergency medicine (EM) ultrasound fellowship-trained physician, as well as at least one EM resident volunteer for every four medical students to facilitate the hands-on portion of the module. There was also one standardized patient for every four medical students. Recruited EM residents had, as an ongoing requirement of their residency, already completed a 2-week training on the use of POCUS. On the day of the module, each EM resident was oriented to the activities and given a facilitation guide (Appendix C) and a checklist of POCUS images to cover (Appendix D). The standardized patients were recruited and prepared in advance (Appendix E). They did not require additional training.

Teaching Activities

1. Didactic session: This PowerPoint-based, 30-minute session consisted of a brief overview of the technology of ultrasonography, general ultrasound techniques, POCUS

Copyright © 2020 Alerhand et al. This is an open-access publication distributed under the terms of the Creative Commons Attribution license.

images of normal anatomy compared to CT scans and cadaver images, and, finally, pathological findings as seen in the physical exam, CT scans, and POCUS (Appendix F). We developed presentation notes to standardize delivery of this presentation for future implementation.

2. Small-group hands-on skills practice: Students were divided into groups of four per room/facilitator for a 1.5-hour hands-on session to practice their POCUS skills. Each group proceeded to a small room and spent 20 minutes with the facilitator and standardized patient assigned to that room. After 20 minutes, the student groups rotated to the next room with a new facilitator and standardized patient. We switched patient and facilitator intentionally to expose students to a variety of patient body types as well as facilitator techniques. Instructors had their own teaching styles and preferred tips on how to obtain an optimal image. Our approach therefore exposed each student to different instructors over the course of the session.

Evaluation

Students completed anonymous pre- and postmodule surveys using statements rated on a Likert scale, as well as free-text responses, to provide feedback on the module and to assess its effects on their learning of the renal system (Appendices G and H). The students were expected (with guidance as needed) to demonstrate the POCUS views listed on their checklist (Appendix D).

Statistical Analysis

The Mann-Whitney *U* test was used to compare the pre- and postmodule survey results.

Results

A total of 31 students signed up for this module, and all interested students were able to participate in it. All completed the pre- and postmodule surveys. There was an overall positive response to the module, with all survey questions seeing an increase in ratings after the module (Table). In particular, there was a statistically significant increase in the degree to which students agreed that the module was relevant to their learning, relevant to their preparation for the USMLE Step 1 Exam, helpful in understanding pathophysiology, and helpful in integrating physical exam learning. There was also a statistically significant increase in the degree to which students agreed they felt comfortable using ultrasound to delineate the anatomy of the renal system, support positive physical exam findings, support other medical imaging tests, visualize the pathophysiology of disease, and make clinical decisions for patients.

The presurvey identified what students had expected to learn from the module. Sample responses included the following:

- “How to perform ultrasound.”
- “Understand anatomy of renal system on ultrasound.”

Table. Student Survey Responses

	Premodule		Postmodule		
**Statement**[a]	***M***	***SD***	***M***	***SD***	**Wilcoxon Rank Sum Test**
My curriculum prepares me well for Step 1.	3.2	0.6	3.3	0.7	.210
I would like ultrasound to be integrated into the medical curriculum.	4.3	0.6	4.5	0.7	.085
An understanding of ultrasound will help me take better care of my patients.	4.5	0.5	4.6	0.5	.224
An understanding of ultrasound will help me in my future career (and specialty).	4.4	0.6	4.6	0.6	.095
The session content ...					
Will allow/allowed for integration of basic science knowledge in a clinical context.	4.2	0.6	4.4	0.6	.082
Will be/was relevant to my learning.	4.3	0.6	4.6	0.5	.015[b]
Met the learning objectives.			4.6	0.5	
Will be/was relevant to my preparation for Step 1.	3.6	0.8	4.0	0.8	.035[b]
The session will help/helped me ...					
Better integrate anatomy learning.	4.4	0.6	4.6	0.5	.075
Better integrate physical exam learning.	4.3	0.6	4.6	0.5	.014[b]
Better understand the use of medical imaging in clinical medicine.	4.5	0.5	4.6	0.6	.257
Better understand pathophysiology of disease.	3.9	0.7	4.3	0.6	.016[b]
Learn to use ultrasound for clinical decision-making.	4.4	0.7	4.6	0.5	.149
I feel comfortable using ultrasound for ...					
Delineating anatomy of the kidneys.	2.2	1.0	4.4	0.6	.001[b]
Supplementing and confirming positive physical exam findings.	2.2	1.1	4.3	0.7	.001[b]
Supplementing and confirming other medical imaging tests.	2.3	1.0	4.2	0.9	.001[b]
Visualizing the pathophysiology of disease.	2.3	1.1	4.1	0.8	.001[b]
Making clinical decisions for my patients.	2.2	1.1	3.7	0.1	.001[b]

[a]Rated on a 5-point Likert scale (1 = *strongly disagree*, 5 = *strongly agree*).
[b]Statistically significant at $p < .05$.

Copyright © 2020 Alerhand et al. This is an open-access publication distributed under the terms of the Creative Commons Attribution license.

- “Learn how ultrasound is used to diagnose renal pathophysiology ... and better understand.”
- “Integration of physical exam findings.”

The postsurvey elicited what students had learned from the module. Sample responses included the following:

- “How to use ultrasound.”
- “When to use ultrasound.”
- “Identify renal system anatomy on ultrasound.”
- “Use ultrasound to visualize and diagnose renal pathology.”
- “How to use ultrasound to make clinical decisions.”
- “The convenience of ultrasound.”

When the students were asked to provide written feedback after the module, their responses were overwhelmingly positive:

- “Informative.”
- “Great session!”
- “Interactive sessions are much more valuable than being lectured on ultrasound.”
- “Integrate ultrasound but make sure it’s hands-on. Very cool. Thank you.”

Discussion

POCUS is one of the most impactful medical innovations of the last decade. It has quickly become the standard of care for bedside clinical decision-making, particularly in urgent-emergent situations. Medical students are being exposed to this tool on clinical rotation. Anecdotally, we have also seen increasing requests from our medical students for earlier exposure to and teaching of POCUS. POCUS as a teaching tool is unique in that it allows for integration of basic sciences such as anatomy with pathophysiology and clinical skills such as physical examination while providing clinical relevance for students in the preclerkship years. The organ systems–based format of these years is also ideal for tailoring POCUS to each organ system. We chose the renal course for our pilot since there was less published content on renal POCUS compared to other organ systems and the imaging is relatively more focused structurally as compared to other systems (e.g., valvular details of a cardiac exam). The timing for planning the module also happened to coordinate well with the upcoming renal system course. Lessons learned have since been applied to other organ systems. We found that it was feasible to implement POCUS to enhance renal organ system teaching for first-year medical students, and the students found POCUS to be a valuable adjunct to their education. Students found this module to be especially helpful in learning general ultrasound technique, indications for ultrasound, renal anatomy and pathophysiology, and correlation of physical exam with disease processes. Based on the pre- and postmodule surveys, the module was able to meet or exceed most of the expectations that students had.

Lessons Learned

Standardized patients: We realized that although our standardized patients had normal anatomy, some had more difficult views of certain images compared to others due to inherent variability in anatomy. Because of this variability, we had students rotate stations so as to be exposed to standardized patients of varying body types. In the future, we plan to scan each standardized patient prior to the module and determine which ones have more favorable views for specific organs.

Time: Balancing the time for didactics versus hands-on components was necessary. We had to ensure that the overview lecture was thorough enough to give students the necessary knowledge to use POCUS and appreciate the significance of renal ultrasound while allotting sufficient time for each student to have supervised hands-on practice. By allotting 30 minutes for the lecture and 1.5 hours for the hands-on session, we were able to achieve our educational objectives. However, some students still felt that the hands-on session was too short. The challenge of scheduling these sessions in a compressed curriculum and balancing both medical students’ and physicians’ schedules and times is an ongoing discussion as we plan to implement POCUS in other organ systems. This will become a bigger challenge as we move from the pilot phase to providing all students in preclerkship years with POCUS experience.

Limitations

Personnel and equipment: While we had access to faculty and residents who were very well trained in POCUS, others may encounter difficulty in finding enough individuals capable of teaching it. However, with POCUS becoming a part of many residency curricula, the recruiting pool to teach this content is easily expandable to intensivists and medicine and surgery disciplines. The literature has shown that instructors of various types and backgrounds, including trained senior medical students, nonclinical anatomists, and electronic programs, can effectively teach POCUS.[2,26-29] Thus, it is possible for individuals to have learned POCUS and to teach it to medical students without having formal advanced training such as radiology residency or ultrasound fellowship. For example, residents learn basic skills in POCUS as part of routine training in EM and other disciplines. Educators wishing to implement this curriculum would also need to have access to at least one ultrasound machine.

Copyright © 2020 Alerhand et al. This is an open-access publication distributed under the terms of the Creative Commons Attribution license.

Number of learners: We limited the number of learners for each module based on the number of ultrasound transducers we had available and the number of EM residents we were able to recruit. We chose to use a resident-to-student ratio of one to four because we felt it would be a good fit for the physical space and time we had for the practical session. This balance of learner to transducer and facilitator will need to be considered and may need to be adjusted based on access to ultrasound machines, facilitators, time, and physical space. For the time, setting, and number of students we had for the module, we felt that the instructor-to-student ratio of one to four was effective in allowing each instructor to provide enough time and attention to each student. We have since used the same ratio for other organ system sessions.

Evaluation of clinical skills: We envisioned this pilot as exposure for this early-level learner rather than as a competency achievement. Therefore, we left it to instructor discretion to determine whether an acquired view was adequate or inadequate. We evaluated students' ability to acquire the POCUS images of interest by having each small-group facilitator subjectively determine each student's ability to acquire POCUS images during the hands-on portion of the module. We received feedback from the small-group facilitators that the majority of students were able to acquire the views of the anatomical structure listed on our OSCE checklist (Appendix D) with little to no assistance. If students did require assistance in acquiring a view, only minor corrections were given, such as changing the angle of the ultrasound transducer or applying more pressure. We recommend that if POCUS competency assessment is the goal of the session, educators explore standardized criteria for image quality assessment.

Recruitment of learners: Since participation in our module was voluntary, it is likely that our selection of learners was biased towards students who were self-motivated to learn POCUS and, thus, more enthusiastic about the module. This may have artificially skewed the students' feedback about our module to be more favorable. To learn about the true efficacy of such content, all learners would need to be included.

Future Directions

Our next steps will be to increase the hands-on time students have by teaching general ultrasound techniques during the hands-on portion of the module instead of during the lecture. Another option is to develop flipped classroom learning material that the students can engage in before the module to allow for more hands-on time and in-person addressing of their queries. We have also started the process of implementing POCUS curricula for the other organ systems–based courses, such as the gastrointestinal, lung, and cardiac courses. Additionally, we plan to explore the effects of these POCUS modules on the students' performances in their organ system courses exam scores, as well as to gauge students' perceptions of whether or not this module helped them with learning course content.

Appendices

A. Hands-on Session Setup Instructions.docx
B. Practical Session Room Setup.docx
C. Hands-on Session Instructor Guidelines.docx
D. OSCE Checklist Renal.docx
E. Note for Ultrasound Models.docx
F. MS1 Renal Lecture With Presenter Notes.pptx
G. Premodule Survey.docx
H. Postmodule Survey.docx

All appendices are peer reviewed as integral parts of the Original Publication.

Stephen Alerhand, MD: Assistant Professor, Department of Emergency Medicine, Rutgers New Jersey Medical School; ORCID: https://orcid.org/0000-0003-2383-0309

April Choi, MD: Resident Physician, Department of Emergency Medicine, Rutgers New Jersey Medical School

Ilya Ostrovsky, MD: Assistant Professor, Department of Emergency Medicine, Rutgers New Jersey Medical School

Sophia Chen, DO, MPH: Assistant Professor, Department of Pediatrics, Rutgers New Jersey Medical School

Christine Ramdin, PhD: Research Associate, Department of Emergency Medicine, Rutgers New Jersey Medical School

Maria Laboy, MD: Administrative Director, Clinical Skills Center, Rutgers New Jersey Medical School

Sangeeta Lamba, MD, MS-HPEd: Professor, Department of Emergency Medicine, Rutgers New Jersey Medical School

Disclosures
None to report.

Funding/Support
None to report.

Ethical Approval
The Rutgers New Jersey Medical School Institutional Review Board approved this study.

Copyright © 2020 Alerhand et al. This is an open-access publication distributed under the terms of the Creative Commons Attribution license.

References

1. Feilchenfeld Z, Kuper A, Whitehead C. Stethoscope of the 21st century: dominant discourses of ultrasound in medical education. *Med Educ*. 2018;52(12):1271-1287. https://doi.org/10.1111/medu.13714
2. Tarique U, Tang B, Singh M, Kulasegaram KM, Ailon J. Ultrasound curricula in undergraduate medical education: a scoping review. *J Ultrasound Med*. 2018;37(1):69-82. https://doi.org/10.1002/jum.14333
3. So S, Patel RM, Orebaugh SL. Ultrasound imaging in medical student education: impact on learning anatomy and physical diagnosis. *Anat Sci Educ*. 2017;10(2):176-189. https://doi.org/10.1002/ase.1630
4. Nelson BP, Hojsak J, Dei Rossi E, Karani R, Narula J. Seeing is believing: evaluating a point-of-care ultrasound curriculum for 1st-year medical students. *Teach Learn Med*. 2017;29(1):85-92. https://doi.org/10.1080/10401334.2016.1172012
5. Rempell JS, Saldana F, DiSalvo D, et al. Pilot point-of-care ultrasound curriculum at Harvard Medical School: early experience. *West J Emerg Med*. 2016;17(6):734-740. https://doi.org/10.5811/westjem.2016.8.31387
6. Davis JJ, Wessner CE, Potts J, Au AK, Pohl CA, Fields JM. Ultrasonography in undergraduate medical education: a systematic review. *J Ultrasound Med*. 2018;37(11):2667-2679. https://doi.org/10.1002/jum.14628
7. Bahner DP, Royall NA. Advanced ultrasound training for fourth-year medical students: a novel training program at The Ohio State University College of Medicine. *Acad Med*. 2013; 88(2):206-213. https://doi.org/10.1097/ACM.0b013e31827c562d
8. Curriculum reports: curriculum structure during pre-clerkship years. Association of American Medical Colleges. Accessed October 16, 2019. https://www.aamc.org/data-reports/curriculum-reports/interactive-data/curriculum-structure-during-pre-clerkship-years
9. Paganini M, Rubini A. Ultrasound-based lectures on cardiovascular physiology and reflexes for medical students. *Adv Physiol Educ*. 2016;40(2):243-247. https://doi.org/10.1152/advan.00010.2016
10. Hammoudi N, Arangalage D, Boubrit L, et al. Ultrasound-based teaching of cardiac anatomy and physiology to undergraduate medical students. *Arch Cardiovasc Dis*. 2013;106(10):487-491. https://doi.org/10.1016/j.acvd.2013.06.002
11. Blackstock U, Carmody K. Transforming learning anatomy: basics of ultrasound lecture and abdominal ultrasound anatomy hands-on session. *MedEdPORTAL*. 2016;12:10446. https://doi.org/10.15766/mep_2374-8265.10446
12. Jones M. Ultrasound of the scrotum. *MedEdPORTAL*. 2010;6:8223. https://doi.org/10.15766/mep_2374-8265.8223
13. Good R, Orsborn J, Stidham T. Point-of-care ultrasound education for pediatric residents in the pediatric intensive care unit. *MedEdPORTAL*. 2018;14:10683. https://doi.org/10.15766/mep_2374-8265.10683
14. Frost D, Nadjafi M, Cavalcanti R. A curriculum for ultrasound-enhanced physical examination rounds. *MedEdPORTAL*. 2013;9:9625. https://doi.org/10.15766/mep_2374-8265.9625
15. Lim SJ, Hayden S, Kritek P, et al. Point-of-care ultrasound for critical care. *MedEdPORTAL*. 2013;9:9619. https://doi.org/10.15766/mep_2374-8265.9619
16. Bollag L, Kent C, Sherman M. Transversus abdominis plane block (TAP block) boot camp. *MedEdPORTAL*. 2014;10:9809. https://doi.org/10.15766/mep_2374-8265.9809
17. Johnson M, Swoboda B. Ultrasound-guided regional anesthesia tutorial. *MedEdPORTAL*. 2012;8:8488. https://doi.org/10.15766/mep_2374-8265.8488
18. Surange RS, Jeygopal NS, Chowdhury SD, Sharma NK. Bedside ultrasound: a useful tool for the on-call urologist? *Int Urol Nephrol*. 2001;32(4):591-596. https://doi.org/10.1023/A:1014421712499
19. Caronia J, Panagopoulos G, Devita M, et al. Focused renal sonography performed and interpreted by internal medicine residents. *J Ultrasound Med*. 2013;32(11):2007-2012. https://doi.org/10.7863/ultra.32.11.2007
20. Lavi A, Tzemah S, Hussein A, et al. A urologic stethoscope? Urologist performed sonography using a pocket-size ultrasound device in the point-of-care setting. *Int Urol Nephrol*. 2017;49(9): 1513-1518. https://doi.org/10.1007/s11255-017-1641-8
21. Nunes AA, Jr Pazeli JM, Rodrigues AT, et al. Development of skills to utilize point-of-care ultrasonography in nephrology practice. *J Bras Nefrol*. 2016;38(2):209-214. https://doi.org/10.5935/0101-2800.20160030
22. Dalziel PJ, Noble VE. Bedside ultrasound and the assessment of renal colic: a review. *Emerg Med J*. 2013;30(1):3-8. https://doi.org/10.1136/emermed-2012-201375
23. Fields JM, Fischer JI, Anderson KL, Mangili A, Panebianco NL, Dean AJ. The ability of renal ultrasound and ureteral jet evaluation to predict 30-day outcomes in patients with suspected nephrolithiasis. *Am J Emerg Med*. 2015;33(10):1402-1406. https://doi.org/10.1016/j.ajem.2015.07.014
24. Wong C, Teitge B, Ross M, Young P, Robertson HL, Lang E. The accuracy and prognostic value of point-of-care ultrasound for nephrolithiasis in the emergency department: a systematic review and meta-analysis. *Acad Emerg Med*. 2018;25(6):684-698. https://doi.org/10.1111/acem.13388
25. Olszynski P, Anderson J, Trinder K, Domes T. Point-of-care ultrasound in undergraduate urology education: a prospective control-intervention study. *J Ultrasound Med*. 2018;37(9):2209-2213. https://doi.org/10.1002/jum.14571
26. Fuchs L, Gilad D, Mizrakli Y, Sadeh R, Galante O, Kobal S. Self-learning of point-of-care cardiac ultrasound—can medical students teach themselves? *PLoS One*. 2018;13(9):e0204087. https://doi.org/10.1371/journal.pone.0204087

Copyright © 2020 Alerhand et al. This is an open-access publication distributed under the terms of the Creative Commons Attribution license.

27. Eroglu O, Coskun F. Medical students' knowledge of ultrasonography: effects of a simulation-based ultrasound training program. *Pan Afr Med J*. 2018;30:122. https://doi.org/10.11604/pamj.2018.30.122.14820

28. Dickerson J, Paul K, Vila P, Whiticar R. The role for peer-assisted ultrasound teaching in medical school. *Clin Teach*. 2017;14(3):170-174. https://doi.org/10.1111/tct.12541

29. Jurjus RA, Dimorier K, Brown K, et al. Can anatomists teach living anatomy using ultrasound as a teaching tool? *Anat Sci Educ*. 2014;7(5):340-349. https://doi.org/10.1002/ase.1417

Received: February 14, 2020
Accepted: August 3, 2020
Published: December 9, 2020

Copyright © 2020 Alerhand et al. This is an open-access publication distributed under the terms of the Creative Commons Attribution license.
